# Man’s Make-Up

**Published:** 1873-01

**Authors:** 


### MAN'S MAKE-UP.

A HEALTHY MAN in the prime of life, weighing about a hundred and fifty or sixty pounds, has the following constituents in the proportions named, the whole making a pail of water and a few pounds of ashes.

| | lb. | oz. | grains. |
|---|---|---|---|
| 1. Silex or sand found in the teeth, nails and hair | | | ½ |
| 2. Magnesia | | | 12 |
| 3. Iron, which colors the blood | | | 100 |
| 4. Potash | | | 290 |
| 5. Fluorine | | 2 | 00 |
| 6. Sulphur | | 2 | 21 |
| 7. Chlorine | | 2 | 47 |
| 8. Salt | | 2 | 116 |
| 9. Phosphorus, the source of vitality which feeds the brain | 1 | 12 | 100 |
| 10. Lime | 2 | 0 | 0 |
| 11. Nitrogen, the foundation of flesh | 3 | 8 | 0 |
| 12. Carbon, supplies animal heat | 21 | 0 | 0 |
| 13. Hydrogen, the basis of water | 14 | 0 | 0 |
| 14. Oxygen, in various combinations, | 111 | 0 | 0 |

The human body is composed of these fourteen elements which are all the time in process of use, are consumed and pass out as wood is consumed in the fire-place, turned into ashes and carried away. Being used, other particles of the same kind must be supplied: that is done by our eating and drinking, and that food which contains all these elements is the most perfect food, although every different article contains different proportions of the elements of which they are composed, and very few contain all the elements necessary to the human body. Wheat, eggs, and milk are, perhaps, the only articles which contain all or nearly all of the elements above mentioned: hence, with plenty of pure water, sunshine, and wheat, life and health can be maintained for a long time; but if a man is fed on sugar or oil exclusively, he would soon begin to pine away, and in a few weeks would die.

### COMPOSITION OF WHEAT.

If each grain of wheat represents a hundred parts, water would be fourteen of these; that is, in one hundred pounds of wheat there are fourteen pounds of water, fat one pound and two-tenths of a pound.

| | lbs. | tenths. |
|---|---|---|
| Fat | 1 | 2 |
| Mineral | 1 | 6 |
| Gum | 1 | 7 |
| Fibre | 1 | 7 |
| Albumen | 1 | 8 |
| Sugar | 5 | 5 |
| Gluten | 12 | 8 |
| Water | 14 | 0 |
| Starch | 59 | 7 |
| | 100 | 0 |

| | lbs. | oz. |
|---|---|---|
| Phosphates | 2 | 0 |
| Nitrate | 14 | 0 |
| Water | 14 | 0 |
| Carbonate | 70 | 0 |
| | 100 | 0 |

The principal constituents of wheat are water to moisten, phosphorus to feed the brain and nerves, nitrate to make flesh, and carbonate to keep warm, to feed the fire of life; the four elements combined have lime enough to make the bones and teeth, in connection with certain elements derived from air and sunshine.

Looking at a wheat grain in another light it contains gluten and starch: the former makes flesh and bones and gives strength to work; the latter gives no strength, no flesh, no bone, but simply warms; hence the most nourishing and strengthening part of wheat is its gluten, the least valuable part is the starch, which, as all know, is very white; the gluten is dark colored; the whitest flour has the least gluten and the most starch, and yet the whiter flour is the higher the price, and the more it is valued as an article for the table. We practically feed the animals with the gluten in the form of bran and eat the starch ourselves. Verily, man is the wisest of animals.

